# Microbiome differences in sugarcane and metabolically engineered oilcane accessions and their implications for bioenergy production

**DOI:** 10.1186/s13068-023-02302-6

**Published:** 2023-03-30

**Authors:** Jihoon Yang, Thanwalee Sooksa-nguan, Baskaran Kannan, Sofia Cano-Alfanar, Hui Liu, Angela Kent, John Shanklin, Fredy Altpeter, Adina Howe

**Affiliations:** 1 Department of Agricultural and Biosystems Engineering, Iowa State University, Ames, IA USA; 2 DOE Center for Advanced Bioenergy and Bioproducts Innovation, Ames, IA USA; 3 Present Address: Agronomy Department, Plant Molecular and Cellular Biology Program, Genetics Institute, University of Florida, IFAS, Gainesville, FL USA; 4 DOE Center for Advanced Bioenergy and Bioproducts Innovation, Gainesville, FL USA; 5 Biology Department, Brookhaven National Laboratory, Upton, NY USA; 6 DOE Center for Advanced Bioenergy and Bioproducts Innovation, Upton, NY USA; 7 Department of Natural Resources and Environmental Sciences, University of Illinois at Urbana-Champaign, Urbana, IL USA; 8 DOE Center for Advanced Bioenergy and Bioproducts Innovation, Urbana, IL USA

**Keywords:** Sugarcane, Metabolic engineering, Endophytic microbiome, Metabolic pathways, Fatty acids, Biosynthesis, Triacylglycerol

## Abstract

Oilcane is a metabolically engineered sugarcane (*Saccharum* spp. hybrid) that hyper-accumulates lipids in its vegetable biomass to provide an advanced feedstock for biodiesel production. The potential impact of hyper-accumulation of lipids in vegetable biomass on microbiomes and the consequences of altered microbiomes on plant growth and lipid accumulation have not been explored so far. Here, we explore differences in the microbiome structure of different oilcane accessions and non-modified sugarcane. 16S SSU rRNA and ITS rRNA amplicon sequencing were performed to compare the characteristics of the microbiome structure from different plant compartments (leaf, stem, root, rhizosphere, and bulk soil) of four greenhouse-grown oilcane accessions and non-modified sugarcane. Significant differences were only observed in the bacterial microbiomes. In leaf and stem microbiomes, more than 90% of the entire microbiome of non-modified sugarcane and oilcane was dominated by similar core taxa. Taxa associated with *Proteobacteria* led to differences in the non-modified sugarcane and oilcane microbiome structure. While differences were observed between multiple accessions, accession 1566 was notable in that it was consistently observed to differ in its microbial membership than other accessions and had the lowest abundance of taxa associated with plant-growth-promoting bacteria. Accession 1566 is also unique among oilcane accessions in that it has the highest constitutive expression of the *WRI*1 transgene. The *WRI*1 transcription factor is known to contribute to significant changes in the global gene expression profile, impacting plant fatty acid biosynthesis and photomorphogenesis. This study reveals for the first time that genetically modified oilcanes associate with distinct microbiomes. Our findings suggest potential relationships between core taxa, biomass yield, and TAG in oilcane accessions and support further research on the relationship between plant genotypes and their microbiomes.

**Supplementary Information:**

The online version contains supplementary material available at 10.1186/s13068-023-02302-6.

## Background

Sugarcane (*Saccharum* spp. hybrid) is the world’s most-produced agricultural commodity in terms of biomass, with a global production of 1.98 billion tons in 2020 [1] and provides the feedstock for 26% of the world’s bioethanol and 80% of the global sugar production [2, 3]. Sugarcane is among the most efficient crops in converting solar energy through C4 photosynthesis into stored chemical energy and biomass [4, 5]. The carbon fixed during photosynthesis is converted into sugar or sugar derivatives and then stored in the stem as a soluble disaccharide, sucrose, which can reach 18% of stem fresh weight in sugarcane cultivars [6, 7].

Modern sugarcane cultivars are derived from crosses between *Saccharum officinarum* (2n = 80, *x* = 10) and *Saccharum spontaneum* (2n = 40–128, x = 8), which contribute to high sugar content and resilience. Breeding efforts to maintain the quality and agronomic performance of elite cultivars have improved sugar yield and quality [8–10], disease resistance [11–15], and ratoon ability [16]. Sugarcane has also been modified through transgenic routes, gene editing, and molecular breeding to improve productivity or its potential as a biofuel crop [17–22]. Specifically, energycane has been engineered to hyperaccumulate lipids in its vegetative biomass through *WRI*1 and *DGAT*1 gene modification [17–20]. These efforts have resulted in metabolically engineered oilcane, which hyper-accumulates energy-dense triacylglycerol (TAG) at levels exceeding the non-modified sugarcane by 30- to 400-fold in vegetable tissues [20, 21]. Production of TAGs and other lipids in high biomass crops has been proposed as a strategy to meet the demands for plant lipid and biodiesel production [17, 21, 22].

In association with plant modifications, research has also targeted the microbial communities associated with sugarcane (i.e., sugarcane microbiomes) for beneficial interactions [23]. Previous efforts to characterize the sugarcane microbiome have shown that stable core taxa exist in the leaf and stem endophytes and soil microbiomes [24]. This core microbiome has been observed to represent less than a quarter of the total observed microbial diversity but comprised the large majority (> 90%) of the relative abundance of the total microbial community. Core taxa have been associated with beneficial plant properties, including *Acidobacteria* and *Klebsiella* [25, 26], associated with plant growth promotion. Known nitrogen-fixing bacteria, *Bradyrhizobium* and *Sphingomonas*, as well as *Burkholderia* have also been identified in the sugarcane microbiomes [27]. While microbiome characterization in sugarcane has been studied, knowledge of the oilcane microbiome has been lacking so far.

In this study, we characterize the microbiomes of three high biomass accumulating oilcane accessions (1565, 1566, and 1569) that constitutively co-express *DGAT*, *OLE*, and the transcription factor *WRI*1 and suppress the TAG lipase *sdp*1 leading to high TAG accumulation of oilcane. These accessions contrast non-modified wild-type (WT) sugarcane and moderately TAG-accumulating 17 T, which only co-expresses *DGAT* and *OLE* [21, 28]. In plant genotypes, WT sugarcane and oilcane accessions differ in global gene and transgene expression, nutrient allocation, and biomass composition. We hypothesize that these differences impact associated microbiomes. For this study, WT sugarcane and oilcane accessions were planted in muck soil derived from a sugarcane production field near Belle Glade, FL, and grown in replicated experiments under controlled environment conditions in a greenhouse. Our results show drastic differences in the microbiomes between plant compartments. Differences both between WT sugarcane and oilcane accessions and between oilcane accessions in the same plant compartments were not as drastic but significant. We discuss the potential implications of our observations for future management of oilcane to enhance lipid and biomass production.

## Results

The microbial community structure of bacteria and fungi was evaluated based on taxa identified from amplicon sequencing of DNA extracted from plant- and soil-associated compartments of greenhouse-grown WT sugarcane and four oilcane accessions. We compared the alpha (within-sample) diversity between accessions. Using the Chao1 (species richness estimator) and Shannon alpha diversity indices, only the bacterial microbiome of one accession, 1566, showed significant differences with the WT sugarcane in the leaf, root, and bulk soil (Additional file 1: Fig. S1). Within leaf-associated microbiomes, the 1566 accession was observed to have increased diversity compared to WT sugarcane (Shannon index. 2.4-fold higher, p_Kruskal–Wallis_ = 0.015). This trend was inversed in root microbiomes, where the 1566 accession was observed to have significantly lower species richness than WT sugarcane (Chao1, 0.3-fold lower, p_Kruskal–Wallis_ = 0.031). In the bulk soil microbiomes, oilcane accessions generally were observed to have higher alpha diversity than WT sugarcane. Among oilcanes, the 1566 accession was observed to be nearly twofold higher in richness (Chao1, p_Kruskal–Wallis_ = 0.034) than WT sugarcane.

We next compared the beta diversity of both bacterial and fungal microbiomes between plant compartments and accessions based on Bray–Curtis dissimilarity. The factor that explained the most variance between the bacterial and fungal microbiomes was the compartment of origin (*R*^2^_PERMANOVA-bacteria_ = 0.665, *p*_PERMANOVA-bacteria_ = 0.001; *R*^2^_PERMANOVA-fungi_ = 0.375, *p*_PERMANOVA-fungi_ = 0.001, Additional file 6: Table S1, Additional file 2: Fig. S2). The bacterial microbiomes between all plant compartments were statistically different (*p*_pairwiseadonis_ < 0.05). Similar results between compartments were observed for fungal microbiomes (*p*_pairwiseadonis_ < 0.05), with the exception of fungal communities from the bulk soil and rhizosphere (*p*_pairwiseadonis_ = 0.07). We also observed a statistically different interaction effect between the accessions and plant compartments in the bacterial microbiomes (*R*^2^_PERMANOVA-bacteria_ = 0.080, *p*_PERMANOVA-bacteria_ = 0.017) but not the fungal microbiomes. Based on these results, the subsequent analysis was focused on the comparisons between WT sugarcane and oilcane accessions within each compartment.

**Fig. 1:**
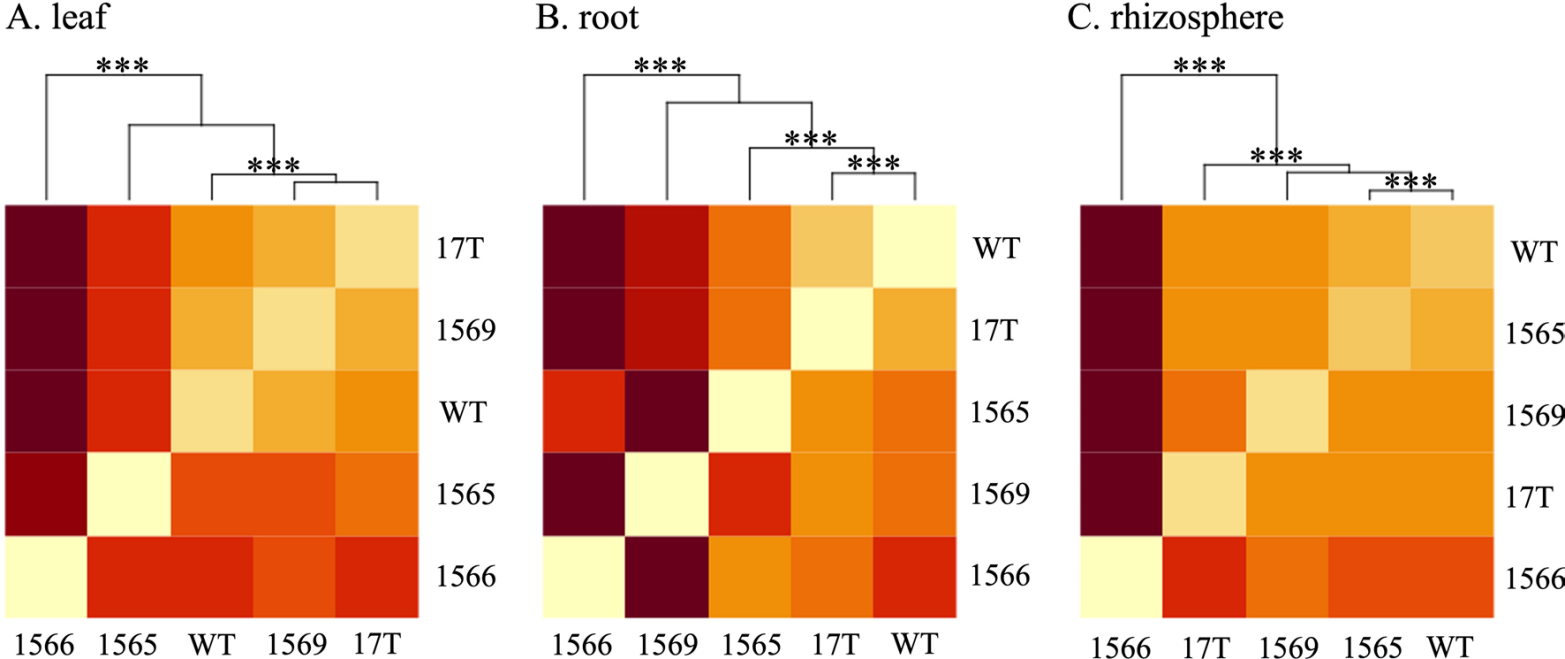
Hierarchical clustering heatmap of Bray–Curtis distance dissimilarity in the bacterial microbiomes. **A** leaf, **B** root, and **C** rhizosphere microbial community composition. The red color indicates a high dissimilarity. Letters *** on the dendrograms denote significant differences in Bray–Curtis dissimilarity indices between accessions at a *p*-value < 0.05 as assessed by hierarchical agglomerative clustering using Ward’s minimum variance method (Ward.D2) with 10,000 bootstrap resampling. WT and 17 T, 1565, 1566, and 1569 represent the wild-type sugarcane and different oilcane accessions, respectively

We observed statistically significant differences between accessions in the leaf, root, and rhizosphere bacterial microbiomes (p_PERMANOVA_ < 0.05). The differences in the fungal microbiome between accessions were observed but not statistically significant (Table 1, Additional file 3: Fig. S3). The largest variation between accessions was observed in the leaf bacterial microbiomes (*R*^2^_PERMANOVA_ = 0.42), followed by the rhizosphere and root microbiomes. While WT sugarcane was observed to be significantly different than oilcane accessions, the observed differences between specific accessions varied depending on the plant compartments. Generally, the 1566 accession and WT sugarcane consistently differed, supported by significant differences in leaf, root, and rhizosphere microbiome (Fig. 1).

**Table 1:** Analysis of variation (PERMANOVA) of microbial community dissimilarity between plant compartments and oilcane accessions

		Bacteria		Fungi	
		R^2^_PERMANOVA_	p_PERMANOVA_	R^2^_PERMANOVA_	p_PERMANOVA_
Leaf	Accession	0.42	0.001	0.26	n.s
	Residual	0.58		0.74	
Stem	Accession	0.20	n.s	0.19	n.s
	Residual	0.80		0.81	
Root	Accession	0.28	0.002	0.22	n.s
	Residual	0.72		0.78	
Rhizosphere	Accession	0.35	0.003	0.31	n.s
	Residual	0.65		0.69	
Bulk soil	Accession	0.24	n.s	0.22	n.s
	Residual	0.76		0.78

**Fig. 2:**
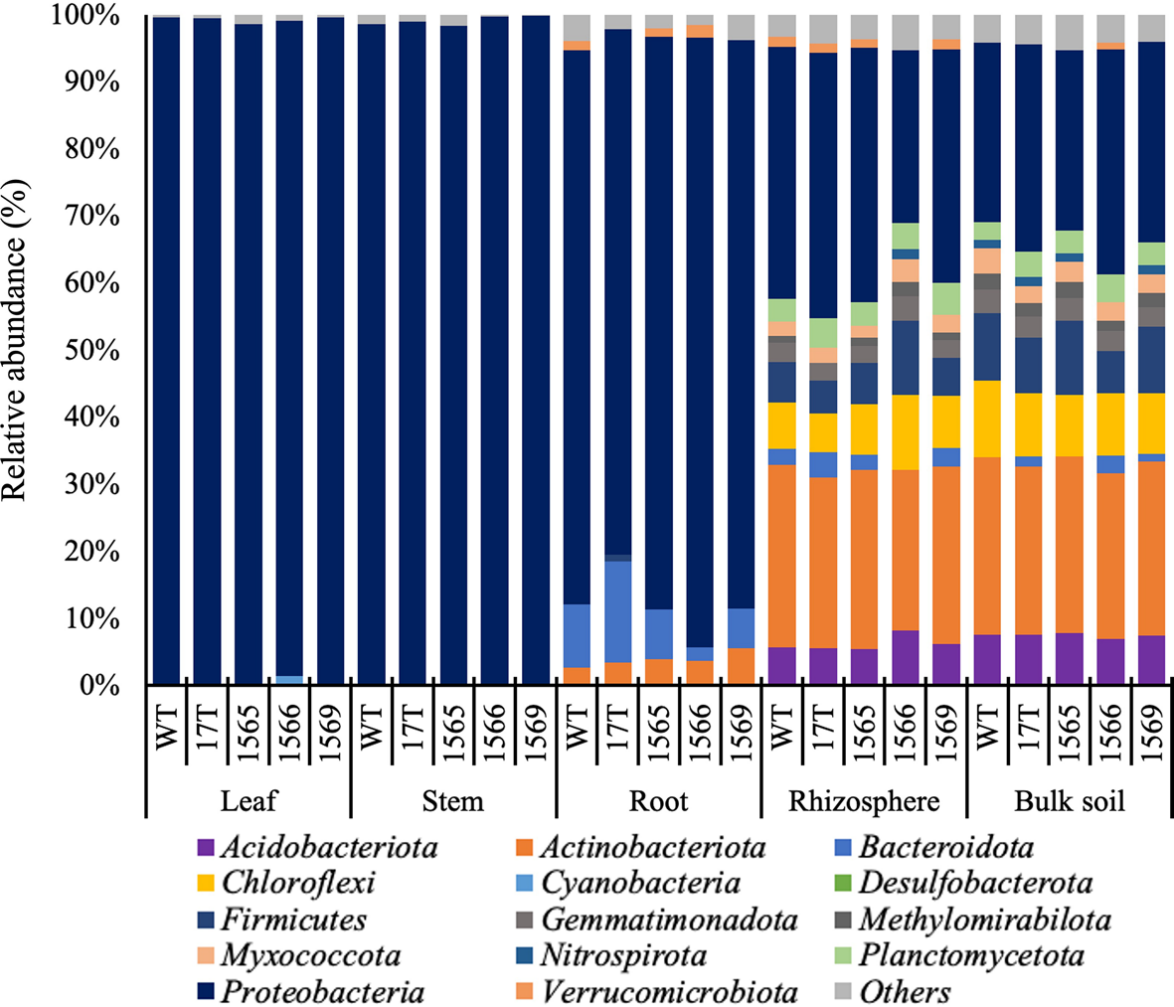
Microbial community composition at phylum level in the microbiomes of WT sugarcane and oilcane accessions. Taxonomic classification of ASVs retrieved at the phylum level using the RDP classifier. WT and 17 T, 1565, 1566, and 1569 represent the wild-type sugarcane and different oilcane accessions, respectively. Others in the legend represent the cumulative relative abundance of taxa with an average relative abundance of less than 1%

To better understand the differences observed between accessions, we next compared the most abundant phyla in the WT sugarcane and oilcane microbiomes. Excluding taxa comprising less than 1% of the total microbial community, we observed a total of 14 phyla. The distribution of these phyla was generally similar between accessions but varied between compartments (Fig. 2). Specific phyla that were significantly different between WT sugarcane and oilcane accessions were identified (Additional file 6: Table S2) and included *Acidobacteriota, Bacteroidota, Chloroflexi, Myxococcota,* and *Proteobacteria* in multiple compartments.

Taxa associated with *Proteobacteria* were dominant in the leaf, stem, and root microbiome of all accessions and were significantly enriched 1.2-fold in the root microbiome of 1566 accession relative to the 17 T accession (p_Kruskal–Wallis_ = 0.023). Given the large representation of bacteria in this phylum, we further evaluated the microbial membership at the genus level. A total of 181 genera associated with *Proteobacteria* were observed in the overall microbiomes, and among them, 38 genera were identified with a greater than 1% average relative abundance. Among these genera, taxa associated with *Cronobacter* were consistently dominant in the leaf and stem microbiomes of all accessions (Additional file 4: Fig. S4).

**Fig. 3:**
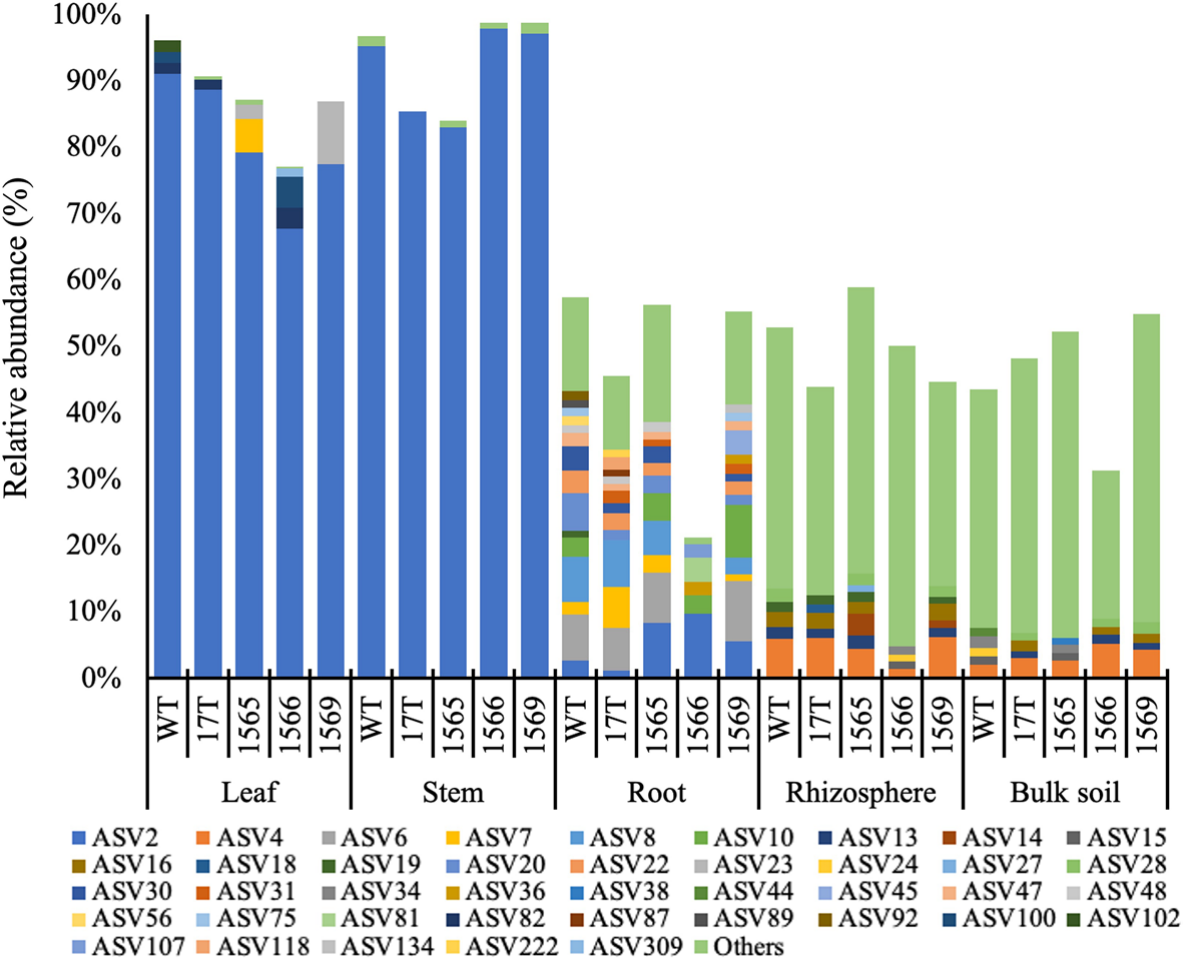
The distribution of core taxa in the microbiomes of oilcane accessions by plant compartment. Core taxa are defined as taxa that are detected at a prevalence greater than 90% in each compartment and accession and also comprise greater than 1% of the relative abundance of the total microbiome. Relative abundances of annotated ASVs are shown, identified to their closest match in the RDP classifier. Leaf, stem, root, rhizosphere, bulk soil represents the origin of microbiomes. WT and 17 T, 1565, 1566, and 1569 represent the wild-type sugarcane and different oilcane accessions, respectively. Others in the legend represent the cumulative relative abundance of taxa with an average relative abundance of less than 1%

To further compare microbiomes between accessions, we characterized their core microbiomes. The concept of a core microbiome has been used previously to characterize trends between human and animal microbiomes [29, 30] and also in sugarcane microbiomes [24, 31]. We define the core microbiome based on the definition proposed by de Souza et al. 2016. The core microbiome included those taxa which are detected at a prevalence greater than 90% in each compartment and accession (e.g., greater than 90% of samples) and comprised greater than 1% of the relative abundance of the total microbiome. A total of 162, 312, 2378, 12,503, and 10,021 ASVs were identified in leaf, stem, root, rhizosphere, and bulk soil microbiomes, of which 7, 8, 92, 543, and 429 ASVs were identified as core taxa (Fig. 3). Overall, core taxa accounted for 3–4% of the total number of observed taxa and up to 99% of the total relative abundance in the microbiomes. We observed a total of five phyla, *Actinobacteriota*, *Bacteroidota*, *Chloroflexi*, *Firmicutes*, and *Proteobacteria*. Among them, *Proteobacteria* was observed in all compartments, and *Actinobacteriota* and *Bacteroidota* were observed in the root, rhizosphere, and bulk soil core microbiomes. *Chloroflexi* and *Firmicutes* were only observed in the core microbiomes of rhizosphere and bulk soils.

Taxa-specific (ASV-based) comparisons of the core microbiomes showed more accession-specific patterns than comparisons of the total microbial community (Figs. 2, 3). A total of 20 core ASVs had significant differences in the relative abundances between accessions in the leaf, root, and rhizosphere microbiomes (Additional file 6: Table S3), and among these, 19 ASVs were associated with *Proteobacteria* and 1 ASV in root microbiomes was associated with *Bacteroidota*. Pairwise comparison of the relative abundance of core taxa between accessions resulted in differences being observed between 1566 and other accessions and WT sugarcane. Statistical differences in relative abundance of core taxa were observed between 1566 and WT, 17 T, 1565, and 1569 in leaves, and between 1566 and WT, 17 T, 1569 in roots. In the rhizosphere (Additional file 6: Table S3), a statistical difference between 1566 and 1565 was observed. Most taxa were present with a relative abundance of less than 5%, but large differences were observed for several taxa in the leaf and root microbiomes. ASV2, associated with the genera *Cronobacter*, was dominant in leaf microbiomes (relative abundance of 68–91%), and we observed that this taxon was most significantly enriched in WT sugarcane relative to the 1566 (relative abundance of 91% in WT sugarcane and 68% in 1566 accession). Other notable core taxa that were observed in specific accessions include taxa associated with the genus *Shinella* (ASV6), *Klebsiella* (ASV7), and *Castellaniella* (ASV8) that were not detected in the 1566 accession.

We next evaluated the metabolic potential of core taxa identified in WT sugarcane and oilcane microbiomes. Functional gene profiles were predicted from the taxonomic profiles of core microbiomes. The composition of the predicted microbial metabolic pathways was generally similar between accessions and between compartments (Additional file 5: Fig. S5). The biosynthesis pathway accounted for ~ 72% of the predicted pathways in the microbiomes, followed by degradation/utilization/assimilation at ~ 19% and precursor metabolites and energy generation pathways at ~ 12%.

**Fig. 4:**
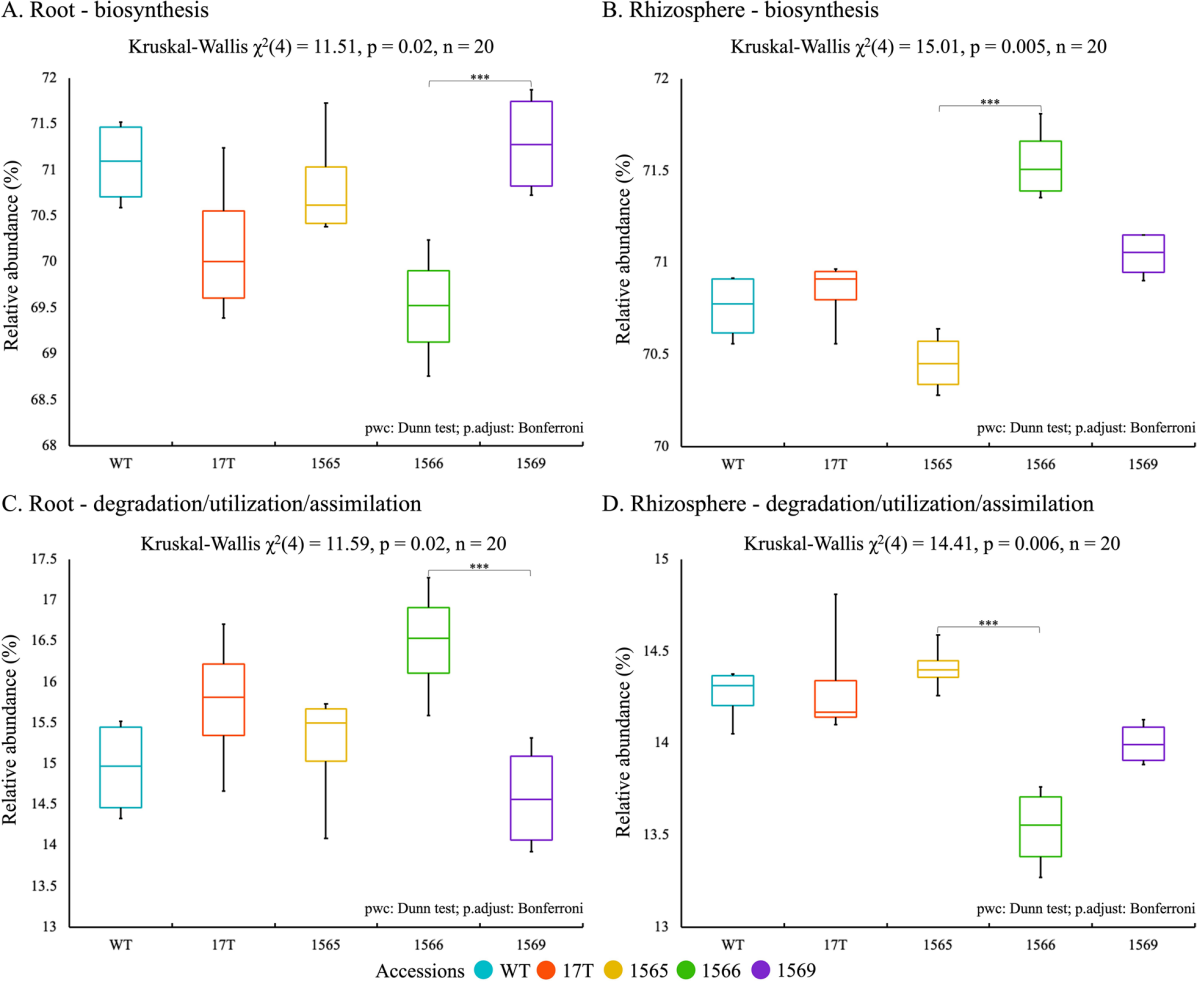
The proportion of significantly different microbial metabolic pathways in the root and rhizosphere core microbiomes. **A** biosynthesis process of root core bacterial microbiome, **B** biosynthesis process of rhizosphere core bacterial microbiome, **C** degradation/utilization/assimilation process of root core bacterial microbiome, and **D** degradation/utilization/assimilation process of rhizosphere core bacterial microbiome. The center horizontal line of the box is the median of the bacterial proportion, the top and bottom of the box are the 25th and 75th quartiles, and the ends of the whiskers are the 5th and 95th quartiles. Letters *** denote significant differences of alpha diversity indices between WT sugarcane and four oilcane accession at a *p*-value < 0.05 as assessed by Kruskal–Wallis with post hoc Dunn’s test. WT and 17 T, 1565, 1566, and 1569 represent the wild-type sugarcane and different oilcane accessions, respectively

Comparing the relative abundance of functions (based on inferred taxa abundances), we observed significant differences in estimated relative abundances of metabolic pathways between oilcane accessions and WT in the root and rhizosphere microbiome (Fig. 4). Significant differences were observed between 1566 and 1569 root microbiomes, where taxa associated with functions related to biosynthesis were enriched in 1569, and degradation, utilization, and assimilation were enriched in 1566. Differences were also observed between rhizosphere microbiomes of 1565 and 1566 in the same functional categories, where taxa associated with biosynthesis were enriched in 1566 and those associated with degradation, utilization, and assimilation were enriched in 1565.

The taxonomic membership of the core microbiome associated with the metabolic functions significantly differed between oilcane accessions (Additional file 6: Table S4). Among the key taxa observed in roots, ASV6 (*Shinella*), ASV7 (*Klebsiella*), ASV8 (*Castellaniella*), ASV10 (*Bordetella*), and ASV89 (*Arachidicoccus*) were highly associated with the biosynthesis pathway. Their relative abundances were highest in 1569 at 20.67% and lowest in 1566 at 2.82%. Among them, ASV6 (*Shinella*) and ASV89 (*Arachidicoccus*) were also related to degradation, utilization, and assimilation pathways, and their relative abundance was the highest at 9.09% in 1569 and was not observed in the core microbiome of 1566. In the rhizosphere, ASV13 (*Bradyrhizobium*) was associated with both the biosynthesis and degradation, utilization, and assimilation pathways. The relative abundance of this taxon was highest in 1565 at 1.92% and was not observed in 1566.

**Fig. 5:**
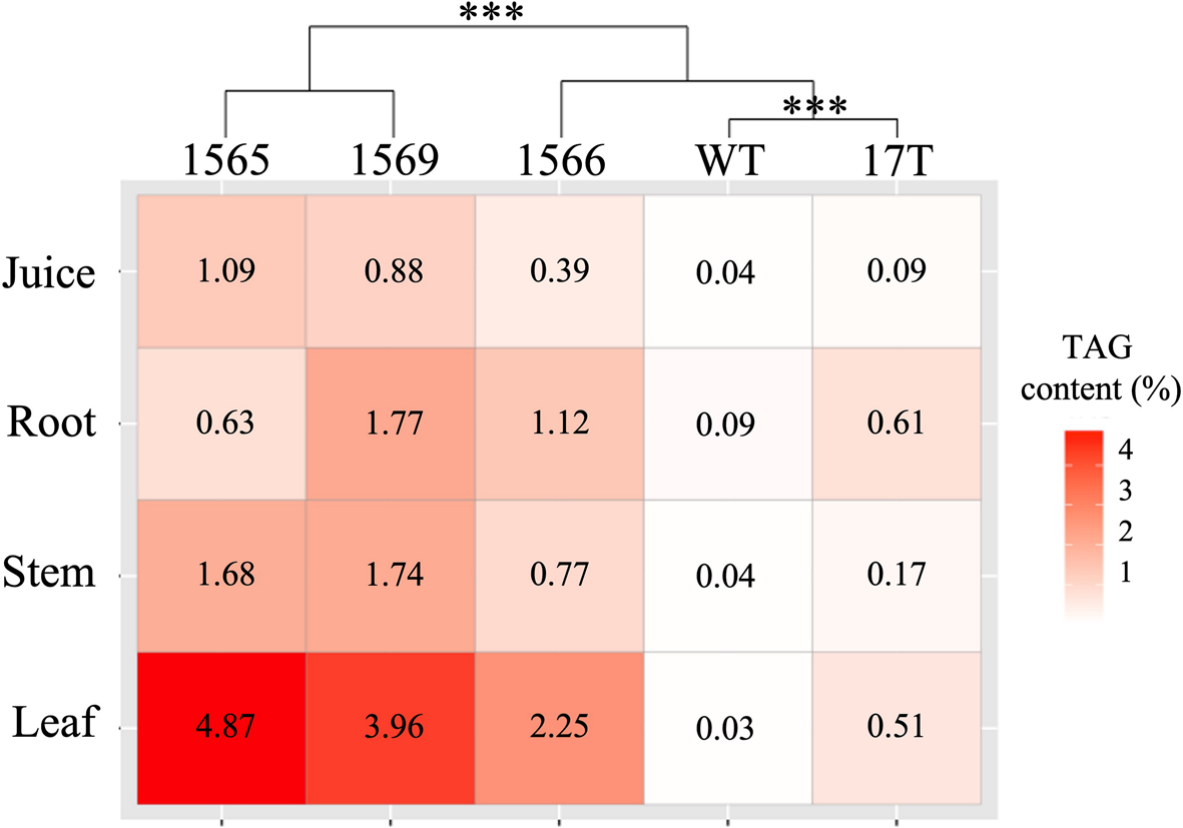
Hierarchical clustering heatmap of TAG content of field-grown WT sugarcane and oilcane accessions. The color scale of the right column bar represents the TAG content. Red indicates a high concentration of TAG, and white indicates a low concentration. Letters “***” on the dendrograms denote significant differences of dissimilarity indices between cultivars at a *p*-value < 0.05

Agronomic performance, TAG accumulation, and transgene expression of oilcane accessions and WT sugarcane were also evaluated under greenhouse conditions (Additional file 6: Table S5, S6). After 10 months of growth in a greenhouse, mean plant height and stem diameter showed a trend of reduction for oilcane accessions compared to WT sugarcane, but this was not statistically significant. The total soluble solids in oilcane accessions did not differ significantly from WT sugarcane (Additional file 6: Table S5). Significant differences between WT sugarcane and oilcane accessions were observed in the number of tillers per plant, juice volume, and TAG contents (Additional file 6: Table S5 and Fig. 5). WT sugarcane had a significantly lower number of tillers per plant than oilcane accessions. Juice volume per unit of stem tissue produced by 17 T and 1566 was significantly lower than the WT sugarcane and other oilcane accessions. TAG accumulation in WT was highest in roots (0.09% of DW), followed by stems (0.04% of DW), juice (0.04% of DW) and leaves (0.03% of DW). Oilcane accessions displayed 7–20 fold the TAG accumulation of WT sugarcane in roots; 4–44 fold the TAG accumulation of WT sugarcane in stems; 2–22 fold the TAG accumulation of WT sugarcane in juice and 17–132 fold the TAG accumulation of WT sugarcane in leaves. Oilcane accessions 1565 and 1569 displayed the highest TAG accumulation in leaves, stems, and juice. Accession 1566 and 1569 displayed the highest TAG accumulation in roots. Accession 17 T accumulated the least TAG among all oilcane accessions but still several fold times than WT sugarcane.

Transgene expression and target gene suppression were determined by quantitative real-time PCR (Additional file 6: Table S6). Oilcane accessions 1565, 1566, and 1569 were harboring transgene *WRI*1, and its expression varied from 0.11 to 0.18 in leaf and 0.23 to 0.24 in stem, with the highest expression detected in accession 1566. 17 T was only transformed with *DGAT*1 and *OLE*1, which were expressed at a higher level as compared to other oilcane accessions. *Sugar dependent*1 (*SPD*1) was suppressed by RNAi in 1565, 1566, and 1569. The percentage of suppression varied from 50 to 75% compared to WT sugarcane.

## Discussion

This study compares, for the first time, the microbiomes of oilcane accessions and non-modified sugarcane. Metabolically engineered oilcane accessions differ in transgene expression and TAG accumulation in the different tissues of the greenhouse-grown plant (Fig. 5 and Additional file 6: Table S6), and these differences are consistent with previous studies [32, 33]. Given the different genetic compositions in oilcanes and non-modified sugarcane, potential differences in their associated microbial communities were explored to provide insights into plant-specific microbial interactions.

Previous studies have characterized the microbiomes of non-modified sugarcane [24, 31, 34–38]. Consistent with previous observations in non-modified sugarcane [24, 31], plant compartments had the greatest effect on the variation of microbiomes in oilcane (Additional file 6: Table S1). We also observed that the leaf and stem microbiomes were similar and that the rhizosphere and bulk soil microbiomes had similar microbial compositions (Additional file 2: Fig. S2). In contrast to other studies that have observed differences in both fungal and bacterial communities [31], we did not observe differences in the fungal communities in any plant compartment. An explanation for this difference is that the oilcanes in this study were grown in natural soil from one location under controlled greenhouse conditions, while the referenced study included sugarcanes grown in different natural soils and environments. An advantage to the design of our study is that it isolates the impacts of accession-specific differences by planting the accessions in the same soils and environment. As future research expands into sugarcane microbiome characterization in field settings, our results will help to distinguish accession-specific impacts from those of soil and the environment. Our results indicate that fungal communities are not significantly impacted by the plant accession, at least for younger sugarcane plants.

Within plant compartments, bacterial communities between accessions were observed to vary significantly, and this was the second largest source of variation in microbial community structure. These results suggest that there are accession-specific bacterial communities that are selected by the plant host. It is likely that these plant-specific microbiomes originate from soil bacteria penetrating plant roots and migrating along their stems to leaves [39–42]. In sugarcane microbiomes, it has been observed that the microbiome comprised a stable population [24, 37, 43, 44], which comprises the large majority (greater than 90%) of its total microbial community. Our results support the presence of stable sugarcane microbiomes, which are similar at the phyla level and comprise the majority of the total microbial population. In our study, this population was generally smaller in oilcane accessions than that of non-modified sugarcane (20–60% vs 90%).

To understand variations between oilcane accessions, we compared compartment-specific microbiomes and taxa that were consistently observed. We used a previously defined concept of a “core microbiome” to identify key taxa. Our results identified that leaf, root, and rhizosphere microbiomes had the largest differences between WT sugarcane and oilcane accessions, consistent with previous studies [45–47]. The greatest deviation from the core taxa of non-modified sugarcane was observed for oilcane accession 1566. Large variations were also observed in the relative abundance of core taxa between oilcane accessions 1566 and other oilcanes. The 1566 accession is characterized by having a higher constitutive expression of the transcription factor *WRI*1 (Additional file 6: Table S6). In field studies, a negative correlation was observed between increased expression of *WRI*1 and oilcane biomass yield [21], and significantly higher biomass accumulation was observed in accessions in which *WRI*1 was not expressed, such as the 17 T used in this study [28]. Constitutive expression of the transcription factor *WRI*1 alters the global gene expression profile, impacting both lipid accumulation and photomorphogenesis with negative consequences for agronomic performance, as recently shown for rice [48]. The observed taxonomic differences between oilcane accessions may potentially be linked to these genetic and phenotypic differences and warrant future functional studies on natural and synthetic plant–microbial interactions in a range of oilcane accessions differing in the level and tissue specificity of transgene expression.

The scope of this study focused on identifying the differences in microbial structure between accessions, but some insight into functional differences can be gained by comparing the functional potential between taxonomic membership. The differences we observed between oilcane accessions were driven by generally prevalent but low-abundant taxa. However, among them, five taxa were identified with greater than 5% relative abundance and significantly different between accessions in the leaf and root microbiomes. Three of these taxa have been previously associated with beneficial plant–microbial functions and include taxa similar to the genera *Cronobacter*, *Klebsiella*, and *Shinella*. Consistent with differences in core taxa distributions, the relative abundance of these taxa in the root microbiome of accession 1566 was different from other oilcanes and non-modified sugarcane and was generally observed to be lower. These genera have been previously characterized to solubilize mineral phosphate while producing indole acetic acid (IAA) [39, 49–53]. Although IAA-producing bacteria are not directly related to TAG accumulation [54, 55], they have been observed to be enriched in the early development of sugarcane, and mainly in buds, shoots, and young leaves [56, 57]. It has previously been shown that biosynthesized IAA is transported through the stem to the roots to support and enhance the growth of plants [58–60]. Thus, the low relative abundance of potentially IAA-producing bacteria observed in the microbiome of 1566 accessions is consistent with previous observations of a decrease in biomass yield compared to WT sugarcane [28].

To explore why the 1566 accession were observed to have such consistent differences in microbiomes relative to other accessions, we compared the genetic modifications between accessions. Oilcane accessions 1565, 1566, and 1569 were transformed with a single multigene construct supporting the constitutive expression of *WRI*1, *DGAT*1-2*, CysOle*1*,* and RNAi suppression of *SDP*1 and *TGD*1. Among them, the *WRI*1 transcription factor, which has previously been correlated to TAG accumulation [28], aids in upregulating the expression of genes involved in plant fatty acid biosynthesis [61] and has been found to affect carbohydrate metabolism by increasing the availability of carbon precursors [62, 63]. Additionally, the constitutive upregulation of this gene has been observed to be correlated positively with decreases in IAA concentration in roots [64] and with reduced biomass accumulation under controlled environments and field conditions [21, 28, 48]. In oilcane accession 1566, we observed higher *WRI*1 gene expression and low relative abundance of plant-growth-promoting bacteria together, and both were also statistically distinct from other oilcane and WT sugarcane (Additional file 6: Table S3). Previously, there has also been a correlation between increased *WRI*1 gene expression and reduction of biomass yield reported [28], further supporting a potential association between the expression of oilcane genes and their microbiomes.

To gain additional insight into functions that may differ between accession-specific microbiomes, we also predicted potential functions represented in observed taxa based on the availability of genomes. Among these functional predictions, we investigated if the relative abundance of IAA production-associated pathways was predicted to be different between accessions. We did not observe significant differences in predicted IAA production between the studied oilcane accessions. Instead, we found differences between root microbiomes of accessions 1566 and 1569 in their predicted functions associated with biosynthesis, including lower abundances of taxa related to *Shinella* and *Castellaniella* in accession 1566. These taxa are known to contribute to the microbial fatty acid synthesis pathways, specifically within the fatty acid synthesis type II (type II FAS) process, in which acetyl coenzyme A is synthesized into long-chain acyl-coenzyme A [65, 66]. We acknowledge that inferred functions based on the presence of taxa and genomes should be used with caution [67, 68], but our results using this approach highlight the opportunity for future research concerning the potential relationships between the functions of specific microbial membership and accession-specific properties.

## Conclusions

This study reveals for the first time that genetically modified oilcanes associate with distinct microbiomes. These differences are consistent across plant compartments and appear to be associated with taxa that have predicted functions related to nutrient cycling and plant growth and development. More broadly, this research supports the general strategy of selecting plant genotypes that promote microbial benefits and plant vigor. Our results highlight the need for a better understanding of the interactions between oilcane genotypes and their microbial communities. Of particular interest is the impact of global gene expression changes mediated by constitutive expression of lipogenic transcription factors like *WRI*1 on the abundance of plant-growth-promoting bacteria. In further developing bioenergy plants, our results support a general strategy to select plant genotypes by exploiting the advantage of microbes.

## Methods

### Sugarcane and oilcane accessions

Sugarcane cv. CP88-1762 was used as a wild-type control and leaf whorl explant donor for the generation of oilcane accessions (17 T, 1565, 1566, 1569) used in this study. Oilcane accession 17 T was co-bombarded with two constructs harboring two linked constitutive expression cassettes of diacylglycerol acyltransferase1-2 (*DGAT*1-2) from *Zea mays* and Oleosin 1 (*OLE*1) from *Arabidopsis thaliana,* respectively, and an unlinked expression cassette of the selectable marker gene neomycin phosphotransferase II (*npt*II) from *Escherichia coli*. Oilcane accessions 1565, 1566, and 1569 were transformed with a single multigene construct containing constitutive expression cassettes of wrinkled 1 (*WRI*1) from *Sorghum bicolor*, *DGAT*1-2 from *Zea mays,* cysteine-oleosin (*CysOle*1) from *Sesamum indicum,* RNAi constructs of sugar-dependent 1 (*SDP*1) and trigalactosyl diacylglycerol 1 (*TGD*1) both from *Saccharum* spp. hybrid, and marker gene *npt*II from *Escherichia coli* as described in Parajuli et al. 2020 [21].

Both oilcane and non-transformed control plants were multiplied by direct organogenesis and rooted plantlets were transferred to 3 L pots containing soil collected from the sugarcane fields in Belle Glade, FL. This soil was from fields in The Everglades Agricultural Area (EAA) at the Everglades Research and Education Center (EREC), Belle Glade, Florida, continuously used for sugarcane production. This Dania muck soil (Histosols) contains approximately 85% organic matter, is high in nitrogen, and low in phosphorus and micronutrient concentrations [69, 70]. Plantlets were acclimatized in a greenhouse while plants were covered with a transparent cup for 5 days to maintain high humidity during the establishment. Greenhouse temperature was controlled with evaporative cooling and ranged between 25 and 30 ℃ during the day and 20 to 25 ℃ during the night.

### Sample collection for microbiome analysis

Plant- and soil-associated samples of WT sugarcane and oilcane accessions were collected after 3 months of cultivation in the greenhouse. Leaf, stem, root, and rhizosphere samples were collected from four biological replicates. For sampling leaves and stems, aerial parts of the plants were separated from the pots using a sterilized pruner. The top four leaves from each of the three stems were combined to get one leaf sample per replicate and the oldest/lower part of three stems from the same plant was pooled to form the stem sample per replicate. The root sample of each plant was separated from the soil by manually shaking the roots onto sterile paper. Using a sterilized pruner, roots were cut and stored separately. After removing large aggregates, rhizosphere was collected in a separate bag. For the bulk soil sample, the original soil that was used for planting was homogenized and a representative soil sample was collected in a separate bag. All these samples were stored immediately on ice and transferred to the − 80 ℃ freezer until further analysis.

### DNA extraction and amplicon sequencing

Endophyte DNA was extracted from the leaves, stems, and roots of the greenhouse-grown plants using a modified previously described procedure [71]. The leaves were washed with distilled water and sterilized with 70% ethanol solution prior to DNA extraction. The stems were similarly washed and subsequently flame sterilized. The roots were separated with a No. 70 USA standard test sieve (pore size 0.212 µm). A distilled water wash was used to remove soil particles, and remaining roots sterilized with 70% ethanol solution. Sterilized leaf, stem, and root samples were separately ground in an ethanol-sterilized blender with phosphate-buffered saline + 0.15% (*v*/*v*) Tween 80. For each sample, the homogenate was horizontally mixed with sterilized beads at 100 rpm at 6 °C for 60 min. The large plant debris was removed by sieving in a sterilized No. 25 USA standard test sieve (pore size 710 µm) and collecting the supernatant in a 50 mL conical tube. The supernatant was centrifuged at 1500 × g for 5 min to remove the small plant debris. For DNA extraction, supernatant was centrifuged at 6500 × g for 10 min. The remaining pellet was collected and re-suspended in 500 µL of nuclease-free water. A total of 200 µL of re-suspended pellet was used for endophyte DNA extraction. For soil samples, soils were homogenized prior to DNA extraction and subsampled to 0.25 g. DNA extraction was performed using the FastDNA Spin Kit and the FastPrep Instrument (MP Biomedicals, USA) for endophyte DNA and DNeasy 96 PowerSoil Pro QIAcube HT (QIAGEN, USA) and QIAcube HT robot (QIAGEN, USA) for soil DNA following the manufacturer’s instructions.

Extracted DNA concentrations were quantified using an Invitrogen Qubit Fluorometer (Invitrogen, USA). DNA sample concentrations above 10 ng ul^−1^ were normalized to 10 ng ul^−1^ prior to sequencing library preparation and samples with concentrations lower than 10 ng ul^−1^ were submitted directly for sequencing library preparation. For bacterial community characterization, the V4 region of the bacterial 16S rRNA gene was amplified with the primers 515F (5′-GTGYCAGCMGCCGCGGTAA-3′) and 806R (5′-GGACTACNVGGGTWTCTAAT-3′) [72, 73]. For fungal community characterization, the ITS3–ITS4 region of the fungal ITS gene was amplified with the primers ITS3 (5′-GCATCGATGAAGAACGCAGC-3′) and ITS4 (5′-TCCTCCGCTTATTGATATGC-3′) [74]. Library preparation and amplicon sequencing was performed on Illumina Miseq with Miseq Reagent Kit V2 (Illumina, USA) at Argonne National Laboratory.

### Amplicon bioinformatics and statistical analysis

The *DADA2* package (version 1.18) in R (version 4.1.0) was used to perform sequencing library quality control and determine the abundance of amplicon sequence variants (ASV) [75]. The Ribosomal Database Project (RDP) Classifier (version 11.5) [76] was used for the taxonomic identification with a confidence threshold of 0.8, and taxa annotation was conducted on the SILVA SSU database release 132 [77] and UNITE database version 8.3 [78] for bacteria and fungi, respectively. PICRUST2 software [79] (http://github.com/picrust/picrust2, version 2.5.0) was used for the metabolic pathway prediction of each observed ASV based on the sequence similarity to the representatives in MetaCyc database [80]. Two alpha diversity indices, Chao1 and Shannon, were used to compare the observed local diversity between WT sugarcane and four oilcane accessions using the *vegan* package (version 2.5–7). Significant differences in alpha diversity between accessions were evaluated using the Kruskal–Wallis test with Dunn’s post hoc test. Bray–Curtis distance dissimilarity based hierarchical clustering was used to compare the beta diversity of bacterial and fungal microbiomes between accessions using the *pvclust* package (version 2.2–0) with Ward’s minimum variance method (Ward.D2) and 10,000 bootstrap resampling. Permutational multivariate analysis of variance (PERMANOVA) was performed with the *adonis* function of the *vegan* package using the Bray–Curtis dissimilarity matrix (version 2.5–7). PERMANOVA was performed to identify significant differences between centroids of each microbiome, with the *R*^2^ statistic represents the proportion of the variance for the separation of the microbiome that was explained by plant compartments or accessions. The comparison between the five (WT sugarcane and four oilcane accessions) groups was accomplished using pairwise PERMANOVA with the *adonis* function of the *vegan* package (version 2.5–7). The level of significance in the statistical analysis was defined as *p* < 0.05 throughout this study.

### Characterization of greenhouse-grown plants

The following phenotypic traits were assessed 10 months after transfer of plants to Dania muck soil. Plant agronomic performance was characterized by the measurement of plant height, number of tillers per plant, stem diameter, juice volume per stem weight, total soluble solids (Brix), and TAG content in compartments. Plant height was measured from the crown to the shoot apical meristem of the main stalk. The number of tillers represents the total number of all tillers per plant. The stem diameter was measured at the middle of the internode using a Vernier caliper. For determining juice volume and total soluble solids, one tiller per accession and replicate was cut, the stem weight was measured after removing leaves and tops, and juice was extracted using a 4-roller sugarcane crusher with two passes of the stems. Total soluble solids were determined using a digital handheld refractometer (PAL-1, ATAGO, Japan). For TAG characterization of plant leaves, stems, roots, and juice, 100–200 mg of leaf tissues from the middle of + 1 (first dewlap) fully expanded leaf were collected in a tube (Eppendorf, USA), lyophilized for two days, and stored at − 80 °C until lipid extraction. For stem tissues, a 1-cm segment of bottom/mature internode sections was collected and ground in a Retsch Cryo mill (Verder Scientific, USA) to get a uniform fine powder. Root samples were washed three times in water and dried in a paper towel to remove excess water. Stem, root, and 10 ml of juice in a conical tube were lyophilized for 3 days prior to lipid extraction. Lipid extraction and TAG determination of WT sugarcane and oilcane tissues were performed by Gas Chromatography–Mass Spectrometry in the Brookhaven National Laboratory, New York, as described in Parajuli et al. 2020 [21]. All agronomic performance analyses were performed in triplicate from each oilcane accession and WT sugarcane.

Additionally, transgene expression was quantified using real-time PCR of *WRI*1, *DGAT*1-2, *OLE*1, *CysOle*1, and *SDP*1. 100 mg of the top fully expanded leaf tissue was collected in a tube (Eppendorf, USA) and flash frozen immediately in the liquid nitrogen from each of the three replications of WT sugarcane and oilcane accessions grown under greenhouse conditions. Total RNA was isolated using TRIzol reagent (Invitrogen, USA) and was treated with RNase-Free RQ1 DNase (Promega, USA) according to the manufacturer’s instructions. First-strand cDNA was synthesized from 500 ng of DNase-treated total RNA using High-Capacity cDNA Reverse Transcription kit (Applied Biosystem, USA). The sugarcane glyceraldehyde 3-phosphate (GAPDH) gene amplicons were used as a reference for the normalization of target gene transcripts as described by Iskandar et al. 2004. Target genes such as *WRI*1, *DGAT*1-2, *OLE*1, *CysOle*1, and *SDP*1 were amplified using gene-specific primers as provided by Parajuli et al. 2020 [21]. Quantitative real-time PCR of the transcripts was performed in the CFX Connect Real-Time PCR (Bio-Rad, USA) with SsoAdvanced SYBR Green Supermix (Bio-Rad, USA) under the following conditions: initial denaturation at 95 °C for 3 min followed by 40 cycles at 95 °C for 10 s and 58 °C for 45 s. Amplification specificity was verified by melt curve analysis and by agarose gel electrophoresis. Relative expression of transgenes and target gene suppressions were calculated using the 2^−ΔΔCt^ method [81]. Analysis of variance was performed for agronomic traits, TAG contents, transgene expression or target gene suppression using Proc GLM and Pearson correlation coefficients between transgene expression or target gene suppression, and TAG contents were determined using Proc CORR implemented in SAS version 9.3 (SAS Institute Inc., USA), as described in Kannan et al. 2022 [28]. The level of significance in the statistical analysis was defined as p_Fisher LSD_ < 0.05 throughout this study.

## Supplementary Information

**Additional file 1: Fig. S1** Alpha diversity indices of bacterial microbiomes. Richness indices (Chao1 and Shannon index) were estimated for microbial communities with ASVs. Letters *** denote significant differences in alpha diversity indices between WT sugarcane and four oilcane accession at a *p*-value < 0.05 as assessed by Kruskal–Wallis with post hoc Dunn’s test. WT and 17T, 1565, 1566, and 1569 represent the wild-type sugarcane and different oilcane accessions, respectively.

**Additional file 2: Fig. S2** Constrained analysis of principal coordinates plots for (A) bacterial and (B) fungal microbiomes. CAP plots were created based on Bray–Curtis distance constrained by compartments (leaf, stem, root, rhizosphere, and bulk soil).

**Additional file 3: Fig. S3** Constrained analysis of principal coordinates plots for bacterial microbiomes by plant compartments. (A) leaf, (B) stem, (C) root, (D) rhizosphere, and (E) bulk soil of WT sugarcane and oilcane accessions. CAP plots were created based on Bray–Curtis distance constrained by accessions (wild-type sugarcane and 17T, 1565, 1566, 1569 oilcane accessions).

**Additional file 4: Fig. S4** Microbial community composition of *Proteobacteria* sub-phyla in the microbiomes. Taxonomic classification of ASVs retrieved at the genus level using the RDP classifier. WT and 17T, 1565, 1566, and 1569 represent the wild-type sugarcane and different oilcane accessions, respectively. Others in the legend represent the cumulative relative abundance of taxa with an average relative abundance of less than 1%.

**Additional file 5: Fig. S5** The composition of the predicted microbial metabolic pathways in the microbiomes. The metabolic pathway prediction of ASVs retrieved at superclass1 level using PICRUST2 software with MetaCyc database. WT and 17T, 1565, 1566, and 1569 represent the wild-type sugarcane and different oilcane accessions, respectively

**Additional file 6: Table S1**. Permutational multivariate analysis of variance for comparing microbial community Bray–Curtis dissimilarity within accessions and compartments. **Table S2**. Average relative abundance of phylogenetic taxa with significant differences in the microbiomes between accessions. **Table S3**. Average relative abundance of core taxa that were significantly different between oilcane accessions. **Table S4**. Core taxa that differ significantly between accessions and their associated microbial metabolic pathways. **Table S5**. Agronomic performance and TAG contents of field-grown WT sugarcane and oilcane accessions. **Table S6**. Expression analysis of lipogenic genes in greenhouse-grown WT sugarcane and oilcane accessions

## Data Availability

The 16S and ITS rRNA sequencing data are available at National Center for Biotechnology Information (NCBI) Sequence Read Archive PRJNA892137.
