# Supplementary figures and images for: Microbiome differences in sugarcane and metabolically engineered oilcane accessions and their implications for bioenergy production
Source: Biotechnol Biofuels Bioprod. 2023 Mar 30;16:56. doi: 10.1186/s13068-023-02302-6 (PMC10064762; doi:10.1186/s13068-023-02302-6)

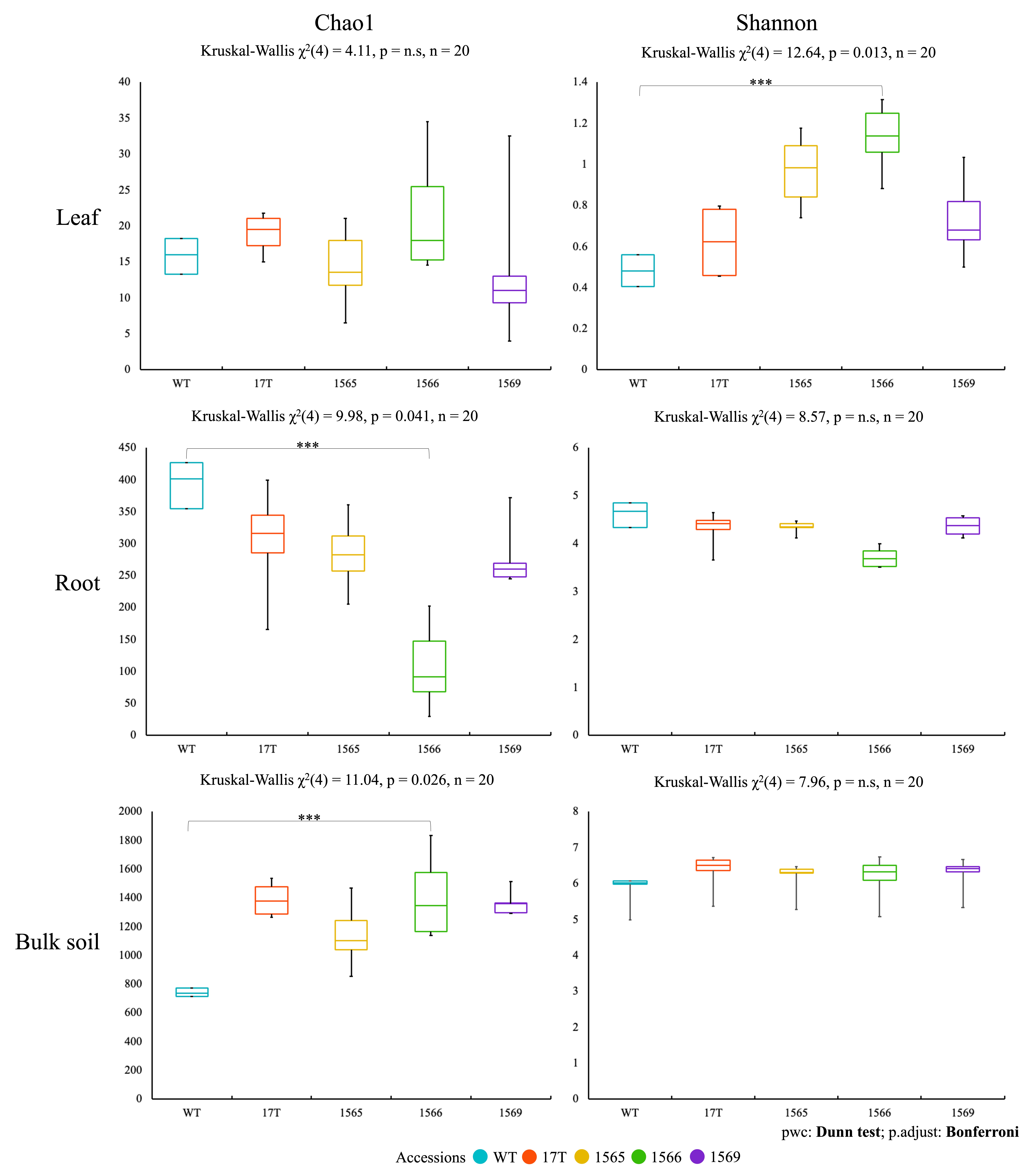

Supplement: Supplementary file 1 — Additional file 1: Fig. S1 Alpha diversity indices of bacterial microbiomes. Richness indices (Chao1 and Shannon index) were estimated for microbial communities with ASVs. Letters *** denote significant differences in alpha diversity indices between WT sugarcane and four oilcane accession at a p-value < 0.05 as assessed by Kruskal–Wallis with post hoc Dunn’s test. WT and 17T, 1565, 1566, and 1569 represent the wild-type sugarcane and different oilcane accessions, respectively. [file 13068_2023_2302_MOESM1_ESM.jpg]

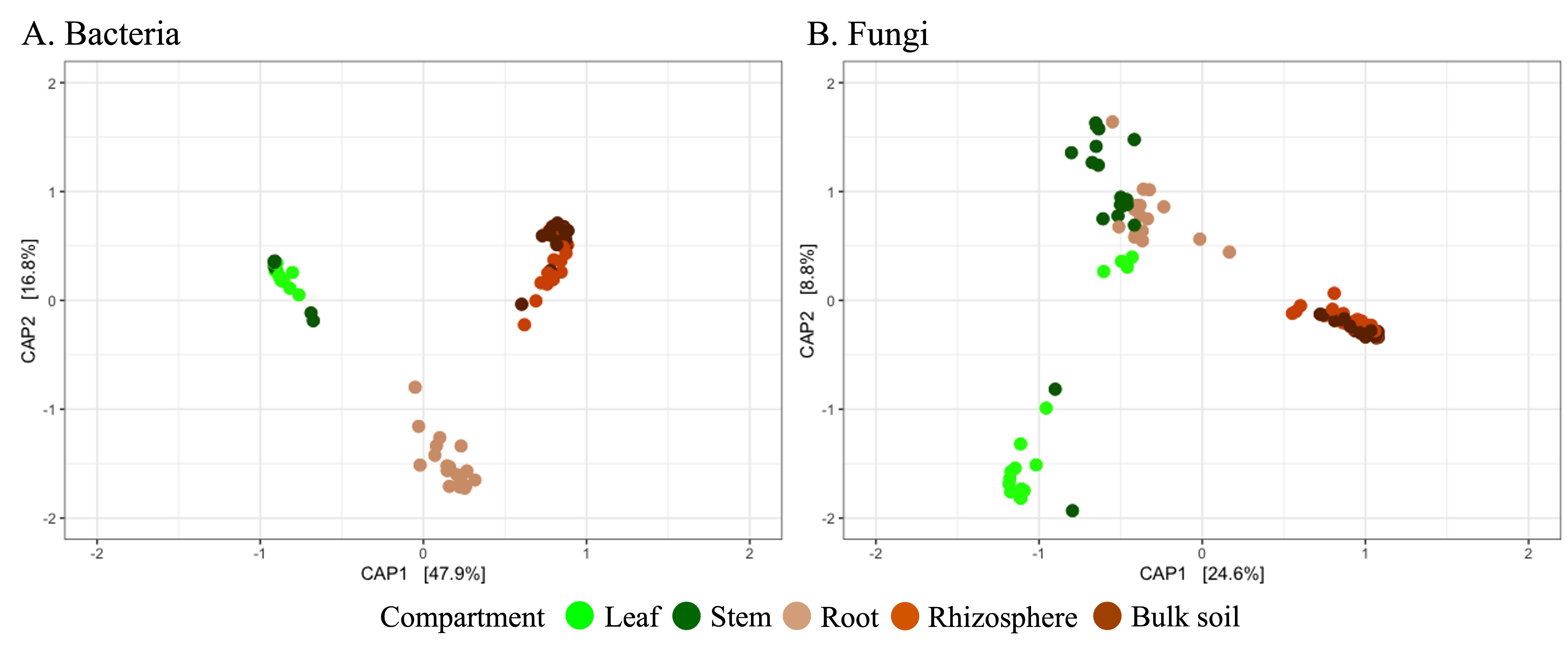

Supplement: Supplementary file 2 — Additional file 2: Fig. S2 Constrained analysis of principal coordinates plots for (A) bacterial and (B) fungal microbiomes. CAP plots were created based on Bray–Curtis distance constrained by compartments (leaf, stem, root, rhizosphere, and bulk soil). [file 13068_2023_2302_MOESM2_ESM.jpg]

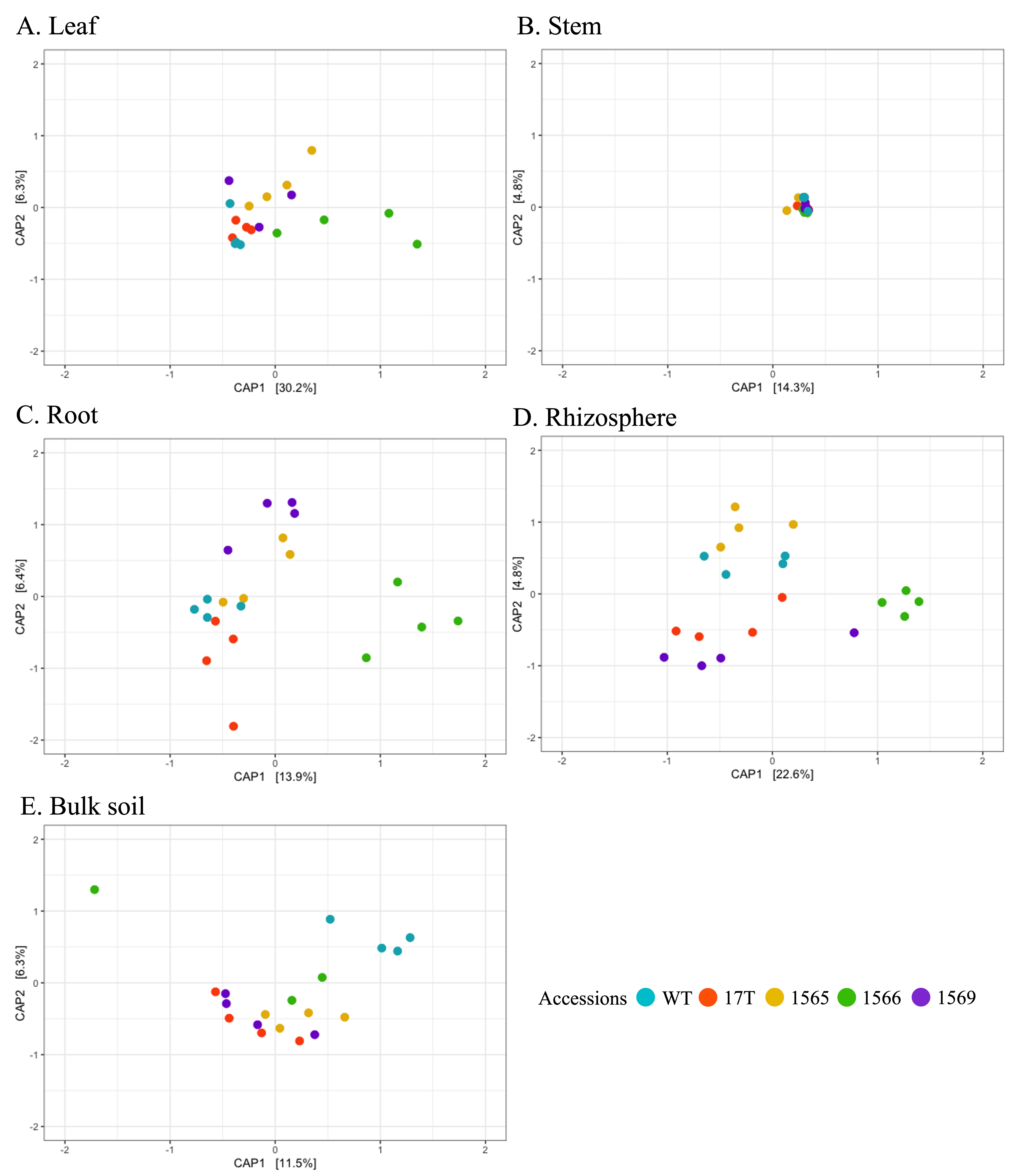

Supplement: Supplementary file 3 — Additional file 3: Fig. S3 Constrained analysis of principal coordinates plots for bacterial microbiomes by plant compartments. (A) leaf, (B) stem, (C) root, (D) rhizosphere, and (E) bulk soil of WT sugarcane and oilcane accessions. CAP plots were created based on Bray–Curtis distance constrained by accessions (wild-type sugarcane and 17T, 1565, 1566, 1569 oilcane accessions). [file 13068_2023_2302_MOESM3_ESM.jpg]

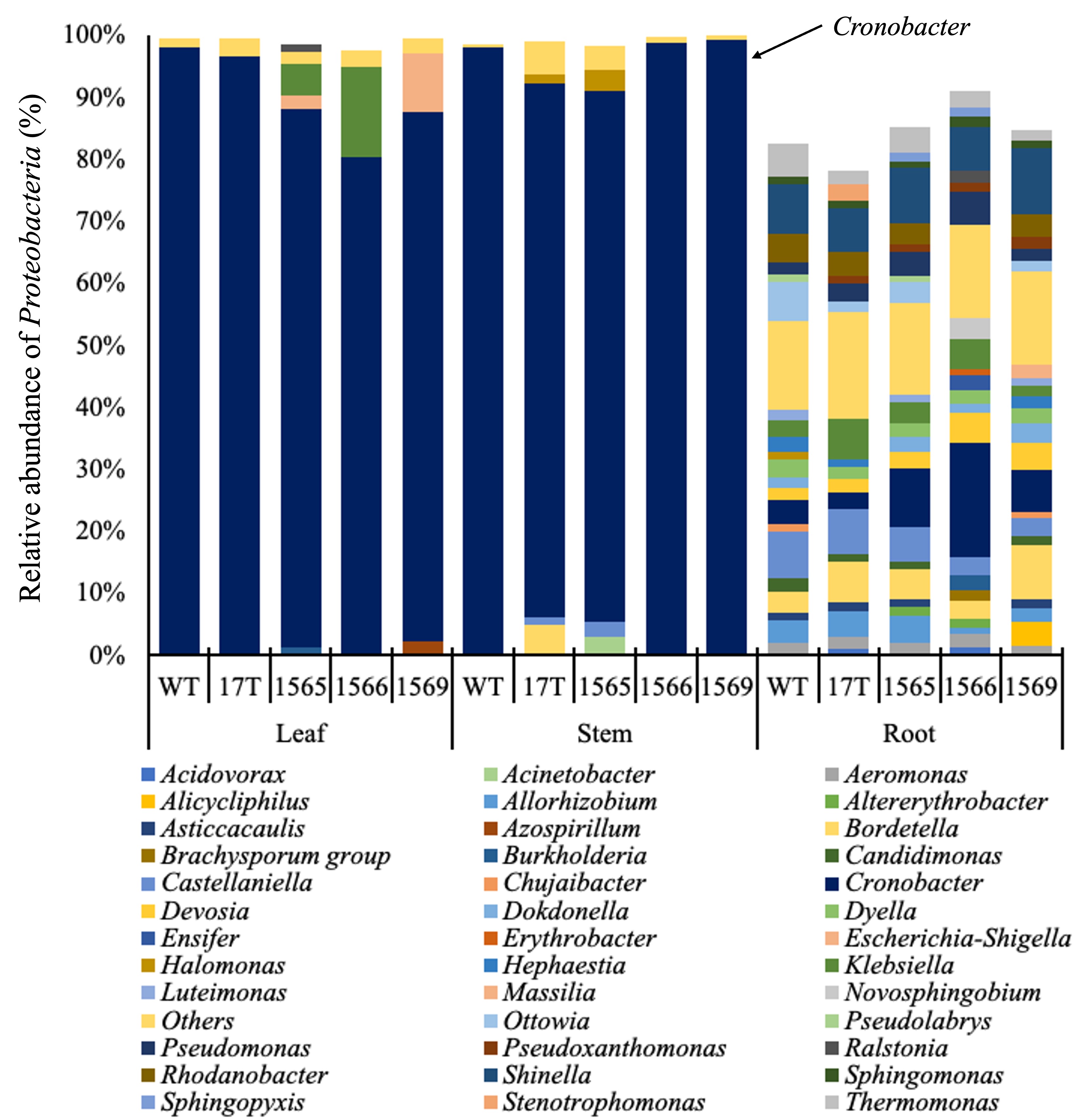

Supplement: Supplementary file 4 — Additional file 4: Fig. S4 Microbial community composition of Proteobacteria sub-phyla in the microbiomes. Taxonomic classification of ASVs retrieved at the genus level using the RDP classifier. WT and 17T, 1565, 1566, and 1569 represent the wild-type sugarcane and different oilcane accessions, respectively. Others in the legend represent the cumulative relative abundance of taxa with an average relative abundance of less than 1%. [file 13068_2023_2302_MOESM4_ESM.jpg]

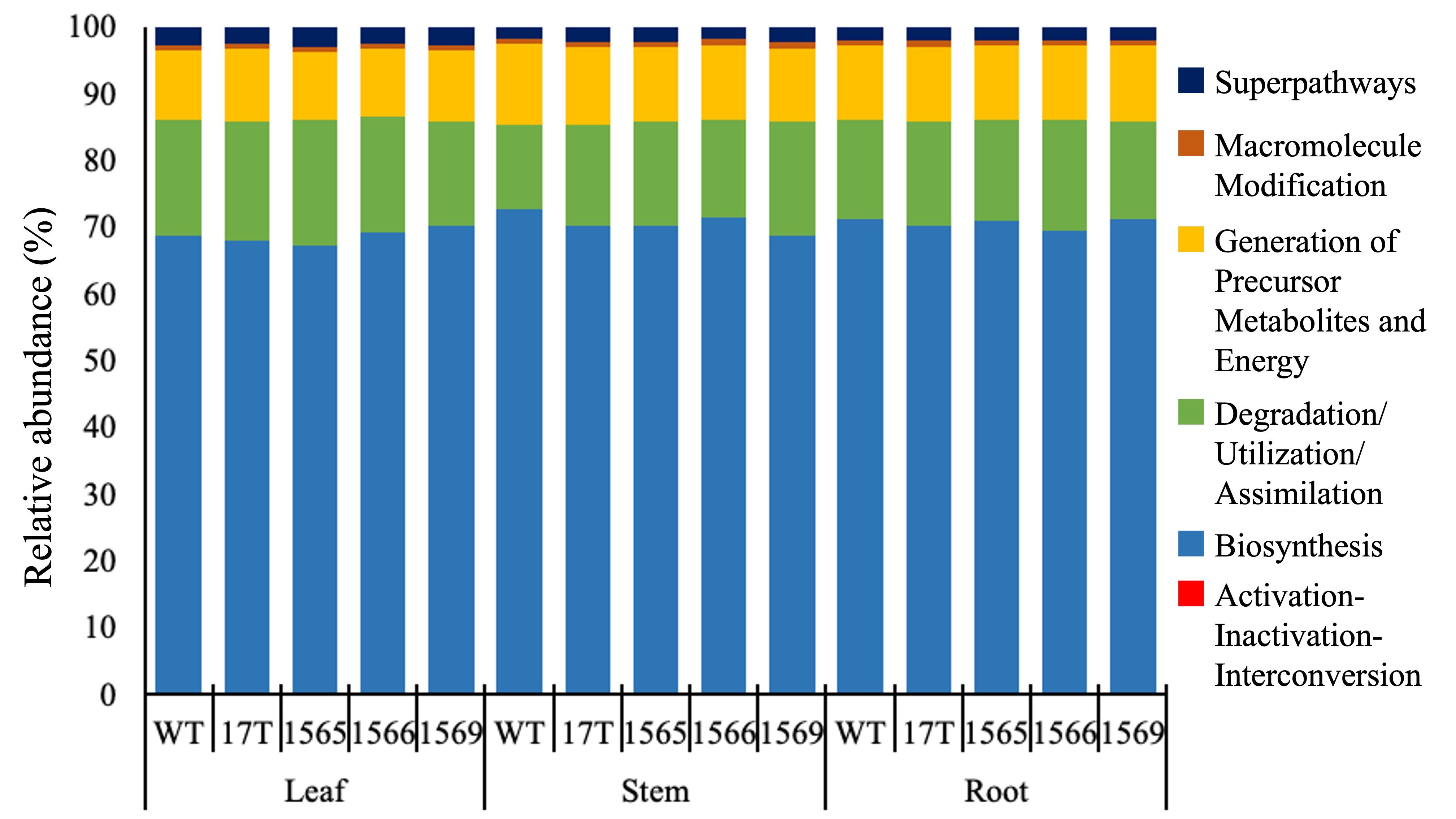

Supplement: Supplementary file 5 — Additional file 5: Fig. S5 The composition of the predicted microbial metabolic pathways in the microbiomes. The metabolic pathway prediction of ASVs retrieved at superclass1 level using PICRUST2 software with MetaCyc database. WT and 17T, 1565, 1566, and 1569 represent the wild-type sugarcane and different oilcane accessions, respectively [file 13068_2023_2302_MOESM5_ESM.jpg]
