# Supplementary material for: Microbiome differences in sugarcane and metabolically engineered oilcane accessions and their implications for bioenergy production
Source: Biotechnol Biofuels Bioprod. 2023 Mar 30;16:56. doi: 10.1186/s13068-023-02302-6 (PMC10064762; doi:10.1186/s13068-023-02302-6)
Supplement: Supplementary file 6 — Additional file 6: Table S1. Permutational multivariate analysis of variance for comparing microbial community Bray–Curtis dissimilarity within accessions and compartments. Table S2. Average relative abundance of phylogenetic taxa with significant differences in the microbiomes between accessions. Table S3. Average relative abundance of core taxa that were significantly different between oilcane accessions. Table S4. Core taxa that differ significantly between accessions and their associated microbial metabolic pathways. Table S5. Agronomic performance and TAG contents of field-grown WT sugarcane and oilcane accessions. Table S6. Expression analysis of lipogenic genes in greenhouse-grown WT sugarcane and oilcane accessions [file 13068_2023_2302_MOESM6_ESM.docx]

Table S1 Permutational multivariate analysis of variance for comparing microbial community Bray-Curtis dissimilarity within accessions and compartments

|  | Bacteria | | Fungi | |
| --- | --- | --- | --- | --- |
|  | R^2^_PERMANOVA_ | p_PERMANOVA_ | R^2^_PERMANOVA_ | p_PERMANOVA_ |
| Accession | 0.018 | n.s | 0.034 | n.s |
| Compartment | 0.665 | 0.001 | 0.375 | 0.001 |
| Accession:Compartment | 0.080 | 0.017 | 0.111 | n.s |
| Residual | 0.237 |  | 0.480 |  |

Leaf, stem, root, rhizosphere and bulk soil microbiota from WT sugarcane, 17T, 1565, 1566, and 1569 oilcane deposits were included

Table S2 Average relative abundance of phylogenetic taxa with significant differences in the microbiomes between accessions

|  | Phylum | Accessions | Relative abundance (%) | | p_kruskal-wallis_ |
| --- | --- | --- | --- | --- | --- |
| Root | *Bacteroidota* | 17T vs 1566 | 15.12  (± 2.36) | 1.95  (± 2.23) | 0.004 |
|  | *Proteobacteria* | 17T vs 1566 | 78.22  (± 3.52) | 90.92  (± 2.70) | 0.023 |
| Rhizosphere | *Acidobacteriota* | 1565 vs 1566 | 5.40  (± 0.39) | 8.17  (± 0.43) | 0.049 |
|  | *Chloroflexi* | 17T vs 1566 | 5.75  (± 2.15) | 11.15  (± 0.48) | 0.041 |
|  | *Myxococcota* | 1565 vs 1566 | 1.76  (± 0.33) | 3.30  (± 0.50) | 0.013 |
| Bulk soil | *Bacteroidota* | WT vs 1566 | 0.65  (± 0.27) | 2.62  (± 1.71) | 0.034 |
|  | *Myxococcota* | WT vs 17T | 3.72  (± 0.28) | 2.58  (± 0.39) | 0.034 |
|  | *Plantomycetota* | WT vs 1566 | 2.75  (± 0.25) | 4.21  (± 0.75) | 0.019 |

The p-value represents the significance assessed by Kruskal-Wallis with post hoc Dunn’s test. WT and 17T, 1565, 1566, and 1569 represent the wild-type sugarcane and different oilcane accessions, respectively

Table S3 Average relative abundance of core taxa that were significantly different between oilcane accessions

|  | Taxa | Genera (Phylum) | Accessions | Relative abundance (%) | | p_kruskal-wallis_ |
| --- | --- | --- | --- | --- | --- | --- |
| Leaf | ASV2 | *Cronobacter*  (*Proteobacter*) | 1566 vs WT | 67.68 (± 10.55) | 91.00 (± 2.04) | 0.015 |
|  | ASV7 | *Klebsiella*  (*Proteobacter*) | 1565 vs WT/17T/1566/1569 | 5.05 (± 3.18) | - | 0.006 |
|  | ASV23 | *Escherichia-Shigella*  (*Proteobacter*) | 1565 vs WT/17T | 2.19 (± 1.52) | - | 0.02 |
|  | ASV100 | *Cronobacter*  (*Proteobacter*) | 1566 vs 17T/1569 | 4.72 (± 1.21) | - | 0.005 |
|  | ASV102 | *Cronobacter*  (*Proteobacter*) | WT vs 17T/1565/1566/1569 | 1.80 (± 1.49) | - | 0.006 |
|  | ASV309 | *Cronobacter*  (*Proteobacter*) | 1566 vs WT/1565/1569 | 1.28 (± 0.81) | - | 0.03 |
| Root | ASV6 | *Shinella* (*Proteobacteria*) | WT vs 1566 | 6.95 (± 3.41) | - | 0.04 |
|  | ASV7 | *Klebsiella* (*Proteobacteria*) | WT vs 1566 | 1.91 (± 1.07) | - | 0.033 |
|  |  |  | 17T vs 1566 | 6.12 (± 4.86) | - | 0.012 |
|  | ASV8 | *Castellaniella* (*Proteobacteria*) | WT vs 1566 | 6.77 (± 2.66) | - | 0.022 |
|  |  |  | 17T vs 1566 | 7.12 (± 2.96) | - | 0.022 |
|  | ASV10 | *Bordetella* (*Proteobacteria*) | WT vs 17T | 2.86 (± 1.74) | - | 0.012 |
|  | ASV19 | *Unclassified* (*Proteobacteria*) | WT vs 1565/1566 | 1.02 (± 0.36) | - | 0.03 |
|  | ASV20 | *Ottowia* (*Proteobacteria*) | WT vs 1566 | 5.73 (± 8.90) | - | 0.022 |
|  | ASV22 | *Rhodanobacter* (*Proteobacteria*) | WT vs 1566 | 3.32 (± 2.56) | - | 0.01 |
|  | ASV30 | *Thermomonas* (*Proteobacteria*) | WT vs 1566 | 3.67 (± 3.46) | - | 0.006 |
|  | ASV45 | *Alicycliphilus* (*Proteobacteria*) | 1569 vs WT/17T/1566 | 3.58 (± 4.87) | - | 0.03 |
|  | ASV47 | *Candidimonas* (*Proteobacteria*) | WT vs 1566 | 1.99 (± 1.24) | - | 0.015 |
|  | ASV48 | *Pseudomonas* (*Proteobacteria*) | WT vs 1566 | 1.23 (± 1.03) | - | 0.022 |
|  | ASV56 | *Dyella* (*Proteobacteria*) | WT vs 17T/1566 | 1.38 (± 0.44) | - | 0.013 |
|  | ASV75 | *Hephaestia* (*Proteobacteria*) | WT vs 1566 | 1.19 (± 0.58) | - | 0.008 |
|  | ASV89 | *Arachidicoccus* (*Bacteroidota*) | WT vs 1565/1566 | 1.16 (± 0.55) | - | 0.016 |
| Rhizosphere | ASV13 | *Bradyrhizobium* (*Proteobacteria*) | 1565 vs 1566 | 1.92 (± 0.43) | - | 0.02 |

The p-value represents the significance assessed by Kruskal-Wallis with post hoc Dunn’s test. WT and 17T, 1565, 1566, and 1569 represent the wild-type sugarcane and different oilcane accessions, respectively

Table S4 Core taxa that differ significantly between accessions and their associated microbial metabolic pathways

| Taxa | Genera (Phylum) | Associated  metabolic pathways | Relative abundance (%) | | | | |
| --- | --- | --- | --- | --- | --- | --- | --- |
|  |  |  | WT | 17T | 1565 | 1566 | 1569 |
| Root |  |  |  |  |  |  |  |
| ASV6 | *Shinella* (*Proteobacteria*) | Amino Acid Biosynthesis | 6.95 (±3.41) | 6.52 (±2.41) | 7.59 (±2.09) | - | 9.08 (±3.97) |
|  |  | Carbohydrate Biosynthesis |  |  |  |  |  |
|  |  | Cell Structure Biosynthesis |  |  |  |  |  |
|  |  | Fatty Acid and Lipid Biosynthesis |  |  |  |  |  |
|  |  | Nucleoside and Nucleotide Biosynthesis |  |  |  |  |  |
| ASV7 | *Klebsiella* (*Proteobacteria*) | Cofactor, Carrier, and Vitamin Biosynthesis | 1.91  (±1.07) | 6.12  (±4.86) | 2.66  (±3.61) | - | 1.03  (±1.26) |
| ASV8 | *Castellaniella* (*Proteobacteria*) | Cell Structure Biosynthesis | 6.77 (±2.66) | 7.12 (±2.96) | 5.16 (±2.45) | - | 2.62 (±1.21) |
|  |  | Cofactor, Carrier, and Vitamin Biosynthesis |  |  |  |  |  |
| ASV10 | *Bordetella* (*Proteobacteria*) | Amino Acid Biosynthesis | 2.86 (±1.74) | - | 4.12 (±2.45) | 2.82 (±1.39) | 7.94 (±5.94) |
|  |  | Cofactor, Carrier, and Vitamin Biosynthesis |  |  |  |  |  |
| ASV89 | *Arachidicoccus* (*Bacteroidota*) | Inorganic Nutrient Metabolism | 1.16  (±0.55) | - | - | - | - |
| Rhizosphere | |  |  |  |  |  |  |
| ASV13 | *Bradyrhizobium* (*Proteobacteria*) | Amino Acid Biosynthesis | 1.73 (±0.42) | 1.28 (±0.31) | 1.92 (±0.43) | - | 1.47 (±0.71) |
|  |  | Aminoacyl-tRNA Charging |  |  |  |  |  |
|  |  | Carbohydrate Biosynthesis |  |  |  |  |  |
|  |  | Cofactor, Carrier, and Vitamin Biosynthesis |  |  |  |  |  |
|  |  | Polyprenyl Biosynthesis |  |  |  |  |  |
|  |  | Aromatic Compound Degradation |  |  |  |  |  |
|  |  | Carbohydrate Degradation |  |  |  |  |  |

WT and 17T, 1565, 1566, and 1569 represent the wild-type sugarcane and different oilcane accessions, respectively

Table S5 Agronomic performance and TAG contents of field-grown WT sugarcane and oilcane accessions

|  | WT | 17T | 1565 | 1566 | 1569 |
| --- | --- | --- | --- | --- | --- |
| Plant height (m) | 2.36  (± 0.13)^a^ | 2.02  (± 0.03)^a^ | 1.88  (± 0.26)^a^ | 1.98  (± 0.05)^a^ | 2.03  (± 0.15)^a^ |
| Number of tillers/plants | 5.00  (± 0.00)^c^ | 10.3  (± 0.88)^a^ | 7.67  (± 0.67)^ab^ | 7.00  (± 1.00)^bc^ | 8.50  (± 0.50)^ab^ |
| Stem diameter (mm) | 17.7  (± 0.70)^a^ | 14.1  (± 0.77)^a^ | 15.9  (± 1.68)^a^ | 16.9  (± 1.55)^a^ | 15.3  (± 0.68)^a^ |
| Juice volume (ml/100 g stem) | 55.5  (± 1.93)^ab^ | 49.7  (± 0.82)^cd^ | 53.8  (± 0.72)^bc^ | 47.3  (± 3.42)^d^ | 59.5  (± 1.17)^a^ |
| Soluble solids (°Brix) | 16.9  (± 1.35)^a^ | 14.3  (± 1.57)^a^ | 17.6  (± 0.97)^a^ | 14.5  (± 0.64)^a^ | 16.8  (± 1.21)^a^ |
| TAG content in leaf (%) | 0.03  (± 0.01)^c^ | 0.51  (± 0.05)^c^ | 4.87  (± 0.81)^a^ | 2.25  (± 0.28)^b^ | 3.96  (± 0.44)^a^ |
| TAG content in stem (%) | 0.04  (± 0.01)^c^ | 0.17  (± 0.02)^bc^ | 1.68  (± 0.31)^a^ | 0.77  (± 0.19)^b^ | 1.74  (± 0.24)^a^ |
| TAG content in root (%) | 0.09  (± 0.00)^b^ | 0.61  (± 0.22)^ab^ | 0.63  (± 0.21)^ab^ | 1.12  (± 0.18)^ab^ | 1.77  (± 0.56)^a^ |
| TAG content in juice (%) | 0.04  (± 0.00)^c^ | 0.09  (± 0.01)^c^ | 1.09  (± 0.13)^a^ | 0.39  (± 0.08)^b^ | 0.88  (± 0.10)^a^ |

WT and 17T, 1565, 1566, and 1569 represent the wild-type sugarcane and different oilcane accessions, respectively. Values are means (n = 3). Means ± standard errors followed by same lower-case letters within the row are not significantly different at p < 0.05

Table S6 Expression analysis of lipogenic genes in greenhouse-grown WT sugarcane and oilcane accessions

|  | WT | 17T | 1565 | 1566 | 1569 |
| --- | --- | --- | --- | --- | --- |
|  | *WRI*1 | | | | |
| Leaf | 0.00  (± 0.00)^c^ | 0.00  (± 0.00)^c^ | 0.11  (± 0.02)^b^ | 0.18  (± 0.02)^a^ | 0.12  (± 0.01)^b^ |
| Stem | 0.00  (± 0.00)^b^ | 0.00  (± 0.00)^b^ | 0.23  (± 0.01)^a^ | 0.24  (± 0.03)^a^ | 0.24  (± 0.03)^a^ |
|  | *DGAT*1-2 | | | | |
| Leaf | 0.00  (± 0.00)^c^ | 1.02  (± 0.18)^a^ | 0.18  (± 0.03)^c^ | 0.47  (± 0.03)^b^ | 0.21  (± 0.01)^bc^ |
| Stem | 0.00  (± 0.00)^c^ | 1.43  (± 0.14)^a^ | 0.20  (± 0.00)^bc^ | 0.43  (± 0.04)^b^ | 0.24  (± 0.01)^bc^ |
|  | *OLE1* | | | | |
| Leaf | 0.00  (± 0.00)^b^ | 2.79  (± 0.10)^a^ | 0.00  (± 0.00)^b^ | 0.00  (± 0.00)^b^ | 0.00  (± 0.00)^b^ |
| Stem | 0.00  (± 0.00)^b^ | 4.07  (± 0.28)^a^ | 0.00  (± 0.00)^b^ | 0.00  (± 0.00)^b^ | 0.00  (± 0.00)^b^ |
|  | *CysOle1* | | | | |
| Leaf | 0.00  (± 0.00)^d^ | 0.00  (± 0.00)^ab^ | 0.01  (± 0.00)^bc^ | 0.02  (± 0.00)^a^ | 0.01  (± 0.00)^c^ |
| Stem | 0.00  (± 0.00)^c^ | 0.00  (± 0.00)^b^ | 0.02  (± 0.00)^b^ | 0.04  (± 0.00)^a^ | 0.02  (± 0.00)^b^ |
|  | *SDP*1 | | | | |
| Leaf | 100  (± 26.0)^a^ | 58.6  (± 3.00)^ab^ | 34.9  (± 3.60)^b^ | 28.5  (± 4.50)^b^ | 50.7  (± 4.90)^b^ |
| Stem | 100  (± 25.9)^a^ | 102.2  (± 13.1)^a^ | 37.3  (± 1.10)^b^ | 33.1  (± 1.30)^b^ | 25.5  (± 0.50)^b^ |

WT and 17T, 1565, 1566, and 1569 represent the wild-type sugarcane and different oilcane accessions, respectively. Values are means (n = 3). Means ± standard errors followed by same lower-case letters within the row are not significantly different at p < 0.05. *WRI*1: *Wrinkled* 1, *DGAT*1-2: *Diacylglycerol acyltransferase* 1-2, *OLE*1: *Oleosin* 1, *SDP*1-RNAi: Inverted repeats of *Sugar dependent* 1
